# Research on Target Detection Based on Distributed Track Fusion for Intelligent Vehicles

**DOI:** 10.3390/s20010056

**Published:** 2019-12-20

**Authors:** Bin Chen, Xiaofei Pei, Zhenfu Chen

**Affiliations:** 1 Hubei Key Laboratory of Advanced Technology of Automotive Components, Wuhan University of Technology, Wuhan 430000, China; chenbinvia@whut.edu.cn; 2 Hubei Collaborative Innovation Center of Automotive Components Technology, Wuhan University of Technology, Wuhan 430000, China; chenzhenfu79@whut.edu.cn

**Keywords:** intelligent vehicle, target detection, MMW radar, camera, sensor fusion

## Abstract

Accurate target detection is the basis of normal driving for intelligent vehicles. However, the sensors currently used for target detection have types of defects at the perception level, which can be compensated by sensor fusion technology. In this paper, the application of sensor fusion technology in intelligent vehicle target detection is studied with a millimeter-wave (MMW) radar and a camera. The target level fusion hierarchy is adopted, and the fusion algorithm is divided into two tracking processing modules and one fusion center module based on the distributed structure. The measurement information output by two sensors enters the tracking processing module, and after processing by a multi-target tracking algorithm, the local tracks are generated and transmitted to the fusion center module. In the fusion center module, a two-level association structure is designed based on regional collision association and weighted track association. The association between two sensors’ local tracks is completed, and a non-reset federated filter is used to estimate the state of the fusion tracks. The experimental results indicate that the proposed algorithm can complete a tracks association between the MMW radar and camera, and the fusion track state estimation method has an excellent performance.

## 1. Introduction

Intelligent vehicles are conducive to reducing traffic accidents and easing traffic congestion, which is an important direction of automobile technology development [1]. Accurate target detection is an important condition for intelligent vehicles to drive normally in the increasingly complex road environment. However, the current sensors used for target detection all have different degrees of defects, such as a limited detection range and poor adaptability to climate and light, leading to incorrect detection information and other problems. In general, a combination of multiple sensors can expand the detection range and improve the detection reliability and robustness [2]. Therefore, sensor fusion technology can be used to solve the problem of target detection.

Currently, the sensors commonly used for target detection on the market include lidar, millimeter-wave (MMW) radar, camera, and ultrasonic radar [3]. Among them, MMW radar can work in all weather, and the detection of distance and speed is relatively accurate. The camera has a wide detection range and target type recognition ability [4]. In addition, the two sensors are cheap. Therefore, the combination of an MMW radar and a camera has become a mainstream scheme for intelligent vehicles.

According to the differences in the data processing methods, sensor fusion can be divided into three levels, namely: data level, feature level, and target level [5]. Data level fusion gathers the original data of each sensor and integrates them on the level of original information to obtain environmental perception results. Feature level fusion extracts the feature information from the original data output by sensors and fusing the feature information. Target level fusion requires each sensor to calculate the position, speed, and contour of the targets according to its own detection information, and to then conduct fusion according to the target information. The data level and feature level generally occur in the target detection stage, while the target level occurs in the target tracking stage. For the fusion of an MMW radar and a camera, because of the large difference of sensor data and high required communication capability, the fusion effect of the data level and feature level is not ideal. Therefore, the target level is more suitable [6].

The key to target level fusion lies in data association and fusion track state estimation [7]. Scholars have done some research on these issues. The authors of [8] used a fuzzy clustering method to associate information from different sensors, and realized fusion estimation based on the parallel filtering method of a centralized fusion structure. The authors of [9,10] developed the weighted track association algorithm to complete the association of local tracks from different sensors, and the cross-covariance fusion algorithm was used to obtain the state estimation of the fusion track. The authors of [11,12] utilized the covariance intersection method to estimate the fusion track state. The authors of [13] adopted the information of different sensors associated with the global nearest neighbor, and obtained a virtual measurement based on the weighted fusion of measurement errors, and carried out the filtering processing. The authors of [14] associated the local tracks of different sensors based on Dempster–Shafer evidence theory, obtained virtual measurements based on maximum likelihood estimation, and processed them with filtering. In the literature [15], a centralized extended Kalman filter is adopted to solve the problem of multi-sensor fusion in single-target tracking. The authors of [16] used the joint probabilistic data association algorithm to complete the association of different sensor tracks and the state estimation of the fusion track. The authors of [17] proposed a new sensor fusion method based on an information matrix, but it did not involve data association.

Through the analysis of the above works of literature, it is known that the current data association methods mostly adopt a single rule. Based on the target information, a statistical value is obtained according to the set rules, and a threshold value is set in advance. The statistical value is compared with the threshold value, to determine whether there is an association. However, in the actual environment, traffic targets are complex and changeable, so it is difficult to select an appropriate threshold to ensure a good association effect. In addition, for the state estimation of the fusion track, there is a problem with a large amount of the calculations. Therefore, based on the above studies, this paper further explores the target level fusion technology of an MMW radar and a camera. This paper designs a fusion algorithm framework based on a distributed structure, and divides the fusion algorithm into two tracking processing modules and one fusion center module. Each tracking processing module is divided into four parts, namely: pretreatment, data association, track management, and state estimation. In the fusion center module, the temporal–spatial alignment is completed, and a two-level association structure combining regional collision association and weighted track association is designed to associate the local tracks that are output by two tracking processing modules. For the fusion tracks, the state estimation is completed based on the non-reset federated filter. Finally, the global tracks’ information obtained by the sensor fusion is output.

The paper is structured as follows, we design the fusion algorithm framework and define different modules in Section 2. Then, Section 3 designs the tracking processing module, and Section 4 designs the fusion center module. Section 5 verifies the feasibility of the proposed algorithm through experiments. Finally, the concluding remarks and future works are presented in Section 6.

## 2. Algorithm Framework

Common processing structures for target level fusion include centralized and distributed structures [18]. The centralized structure has only one data processing module, which is also the fusion center. The measurement information detected by each sensor is transmitted to the fusion center, which associates the fusion track, and the measurement also updates the fusion track state. The distributed structure has multiple tracking processing modules and a fusion center module. The measurement information of each sensor is transmitted to the corresponding tracking processing module, and the tracking processing module outputs the local tracks of the sensor. The local tracks of each sensor enter the fusion center, and the fusion center processes the local tracks and obtains the global tracks, which are the final result of the fusion algorithm.

**Figure 1:**
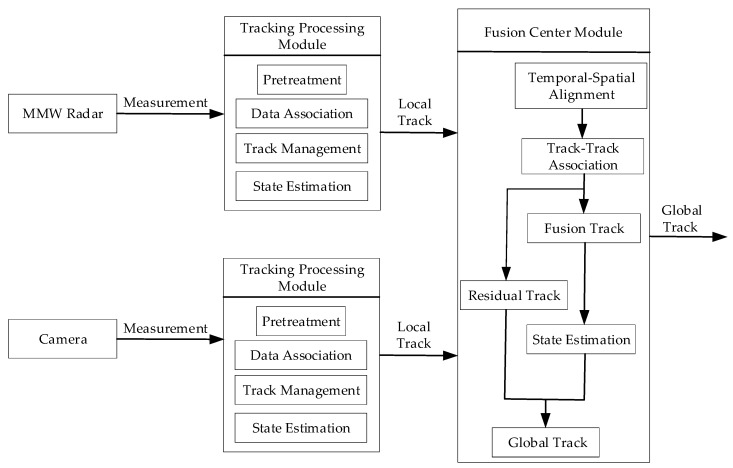
Fusion algorithm framework. MMW—millimeter-wave.

Compared with the centralized structure, the distributed structure has a good stability and low requirements on the communication ability and computing speed of the system. Therefore, the distributed structure is selected as the basic structure of the fusion algorithm in this paper, and the designed fusion algorithm framework for an MMW radar and a camera is shown in Figure 1. The framework divides the fusion algorithm into two tracking processing modules and one fusion center module. The tracking processing module receives the sensor measurement information and carries out multi-target tracking processing, which is divided into several parts, including pretreatment, data association, track management, and state estimation. The fusion center module is mainly divided into several parts, including temporal–spatial alignment, data association, fusion track state estimation, and global track generation. When the system works, the MMW radar and camera separately output measurement information through the CAN communication, and the measurement information enters their respective tracking processing modules. The tracking processing module runs a multi-target tracking algorithm and outputs local track information, including track state and state covariance, which will enter the fusion center module. The fusion center module first registers the local tracks of two sensors in time and space, and then makes the association between the local tracks. The local track is divided into two parts through the track–track association algorithm. One part is the successfully associated tracks, which is called the residual track, and its state information is the same as that of the local track. The other part is the successfully associated tracks, which generate the fusion track, and then estimate the state of the fusion track according to the corresponding local track. The fusion track and residual track constitute the global track, which is the output information of the algorithm framework. Section 3 and Section 4 will design the tracking processing module and the fusion center module, respectively.

## 3. Tracking Processing Module

**Figure 2:**
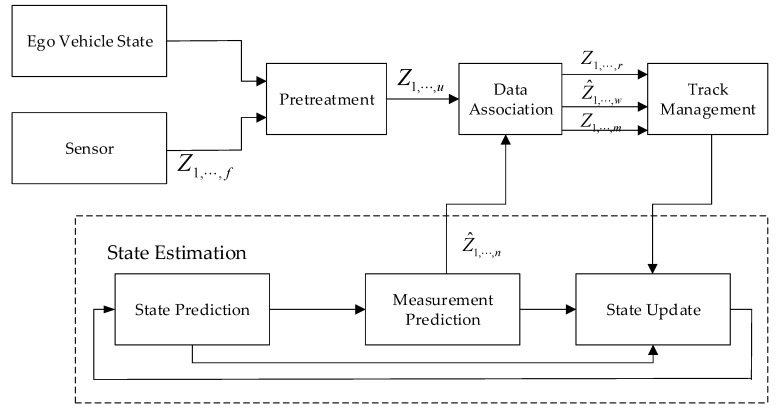
Tracking processing module.

Figure 2 shows the algorithm structure of the tracking processing module. After the measurement information is entered into the tracking processing module, the measurement targets that influence the running of the ego vehicle are firstly selected by combining with the status information of the ego vehicle, and pretreatment is completed. Then, the measurement is associated with the current local tracks. We assume that there is a local track, *i*, and the measurement prediction is $z_{i}\left(k|k-1\right)$ at time, *k*, defined here:

(1) $$d_{ij}^{2}\left(k\right)={\left[z_{i}\left(k|k-1\right)-z_{j}\left(k\right)\right]}^{T}S_{ij}^{-1}[z_{i}\left(k|k-1\right)-z_{j}\left(k\right)],$$

where $z_{j}\left(k\right)$ is the value of measurement target *j*; $S_{ij}$ is the covariance matrix of innovation; and $d_{ij}^{2}$ is the weighted norm of innovation vector, which can be understood as the statistical distance between the measurement prediction information of the local track and the measurement target.

The statistical distance is taken as the association reference, and the Kuhn–Munkres algorithm is taken as the allocation reference, and the association between track and measurement is completed according to the global nearest neighbor idea [19]. The relationship between the measurement and track after the association is completed can be divided into three categories, namely: if the measurement and track are successfully associated, the track is not associated with any measurement, and the measurement is not associated with any track. These association results will be fed into the track management section.

The main function of track management is to manage the generation, maintenance, and disappearance of the track. Track management can solve the problem of false measurement and missing target detection [20]. The measurement target that is not associated with any track is a generated temporary track. For the continuous multi-frame successfully associated temporary track, what can be considered as a real and confirmed track is generated, which is also local track. For the confirmed track, if there is no association measurement in continuous multiple frames, the track can be considered dead and discarded [21]. After the rule judgment, it is necessary to update the status of the confirmed track and temporary track to obtain the optimal state estimation. For the track that is determined to be dead, it is deleted from the track list without any state update.

State estimation is divided into state prediction, measurement prediction, and state update, which is the same as the Kalman filter [22]. Assuming that the acceleration of the target is constant in a short time, a motion model with constant acceleration can be established. The target state vector is

(2) $$X={\left[x,\dot{x},\ddot{x},y,\dot{y},\ddot{y}\right]}^{T},$$

where (x,y) is the position vector, $\left(\dot{x},\dot{y}\right)$ is the velocity vector, and $\left(\ddot{x},\ddot{y}\right)$ is the acceleration vector. Then, the motion state model can be obtained

(3) $$X(k+1)=diag\left[F_{CA},F_{CA}\right]X(k)+diag[\left[G_{CA},G_{CA}\right]W(k),$$

where $F_{CA}=\left[\begin{array}{ccc} 1 & T & \frac{T^{2}}{2}\\ 0 & 1 & T\\ 0 & 0 & 1 \end{array}\right]$, $G_{CA}=\left[\begin{array}{c} \frac{T^{2}}{4}\\ \frac{T}{2}\\ 1 \end{array}\right]$. $W=\left[\begin{array}{c} w_{x}\\ w_{y} \end{array}\right]$ is the white noise sequence in a discrete model, and $w_{x}$ and $w_{y}$ correspond to the target’s noise “jerk” along the x- and y-axis, respectively.

The MMW radar can detect the target distance, azimuth, and relative velocity, and its measurement vector be expressed as follows

(4) $$Z_{r}={\left[\begin{array}{ccc} S & \theta & v \end{array}\right]}^{T}.$$

The corresponding measurement model is as follows:

(5) $$z_{r}(k)=h(X(k))+\upsilon_{r}(k)=\left[\begin{array}{c} \sqrt{x^{2}+y^{2}}\\ \arctan\frac{y}{x}\\ \frac{x\dot{x}+y\dot{y}}{\sqrt{x^{2}+y^{2}}} \end{array}\right]+\upsilon_{r}(k),$$

where $\upsilon_{r}={\left[\begin{array}{ccc} \upsilon_{S} & \upsilon_{\theta } & \upsilon_{v} \end{array}\right]}^{T}$ represents the measurement white noise sequence. Because of the nonlinearity of the measurement model, an extended Kalman filter is used to estimate the track state of the MMW radar.

A camera can detect the target distance, and its measurement vector can be expressed as follows

(6) $$Z_{c}={\left[\begin{array}{cc} x & y \end{array}\right]}^{T}.$$

The corresponding measurement model is as follows:

(7) $$z_{c}\left(k\right)=H_{c}X\left(k\right)+\upsilon_{c}(k),$$

where $\upsilon_{c}={\left[\begin{array}{cc} \upsilon_{x} & \upsilon_{y} \end{array}\right]}^{T}$ represents the measurement white noise sequence. $H_{c}$ is the measurement matrix

(8) $$H_{c}=\left[\begin{array}{cccccc} 1 & 0 & 0 & 0 & 0 & 0\\ 0 & 0 & 0 & 1 & 0 & 0 \end{array}\right].$$

Both the motion state model and the measurement model are linear, so the track state of the camera is estimated through a linear Kalman filter.

## 4. Fusion Center Module

In the fusion center module, the camera detection cycle is taken as the fusion time node. In other words, during the camera detection cycle, the fusion center module will start to run after the camera tracking processing module is completed. First, the local track information of the two sensors is transformed into the same coordinate system to ensure spatial registration. In this paper, the motion coordinate system of the MMW radar is used as the fusion track coordinate system, so we only need to convert the local track information of the camera, which only involves to the conversion of a two-dimensional cartesian coordinate system, which will not be described in detail here. Moreover, every time the camera outputs a set of CAN messages, the track information obtained from the MMW radar in the previous N-cycles is fitted with the quadratic curve according to the least square method. Then, the fitted curve is extrapolated to the current time node of the camera in order to obtain the estimated value of the MMW radar track information [23]. The temporal–spatial alignment is completed. The following parts mainly design the track–track association and fusion track state estimation.

### 4.1. Track–Track Association

#### 4.1.1. Association Algorithm Design

The measurement information of the two sensors is processed by their respective tracking processing modules in order to obtain effective targets, namely a local track. Intuitively, if the effective targets of the two sensors are close enough together, the two targets can be considered to be associated. This is an association method based on the location threshold. Specific to the MMW radar and the camera used in intelligent vehicles, in general, the longitudinal distance measurement of the MMW radar is relatively accurate, the lateral distance measurement is relatively rough, while the camera is just the opposite. This leads to a large deviation between the position of the MMW radar target and the camera target, and the greater distance between the target and the ego vehicle, the greater the deviation.

It is difficult to get good association results only depending on the position threshold, so the motion state information of the target can be further considered. The tracking processing module outputs the local track information, which can be used to compare the similarity of the motion state between the different sensor targets. The track information is used to determine the degree of association, and commonly-used methods include weighted track association [24], etc. However, when the environment is complex and there are many targets, the correlation performance of the weighted track association method will decrease, and there will be many errors and omissions in the associated track.

**Figure 3:**
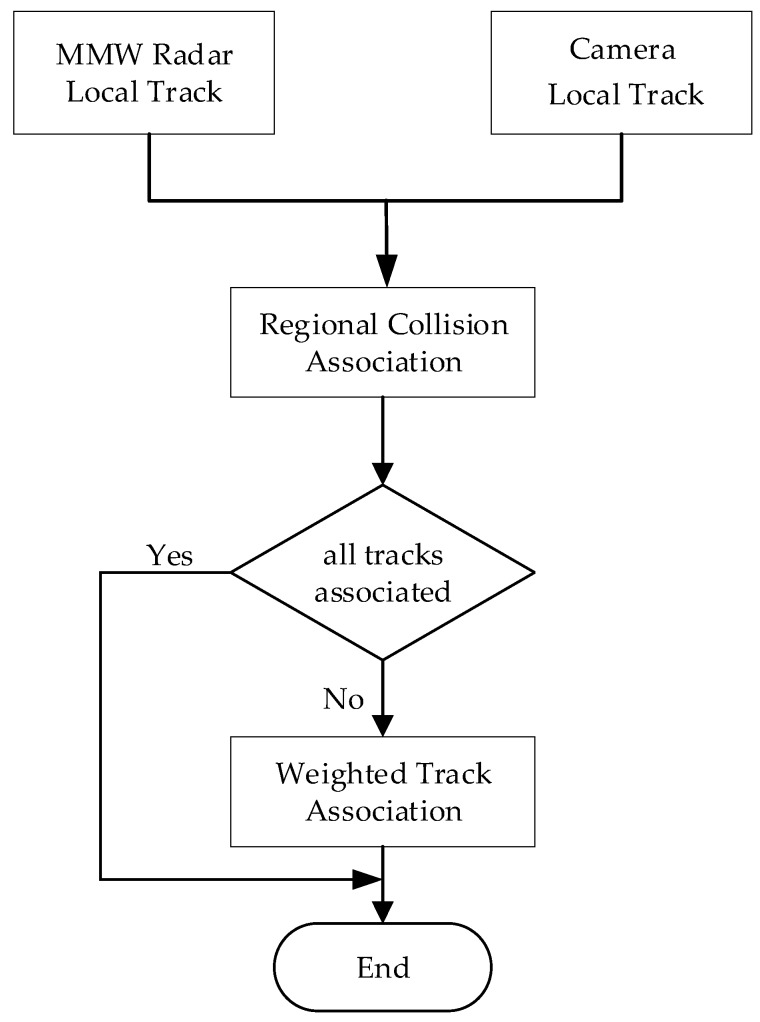
Two-level association structure.

After comprehensive consideration, this paper designs a two-level association structure, as shown in Figure 3. Firstly, the regional collision association algorithm is designed based on the idea of a location threshold. Then, the unassociated local tracks are input into the weighted track association part. Because one association has been passed, the number of targets that need to be associated is decreased, the environmental complexity is reduced, and the weighted track association can play a better role.

#### 4.1.2. Regional Collision Association

**Figure 4:**
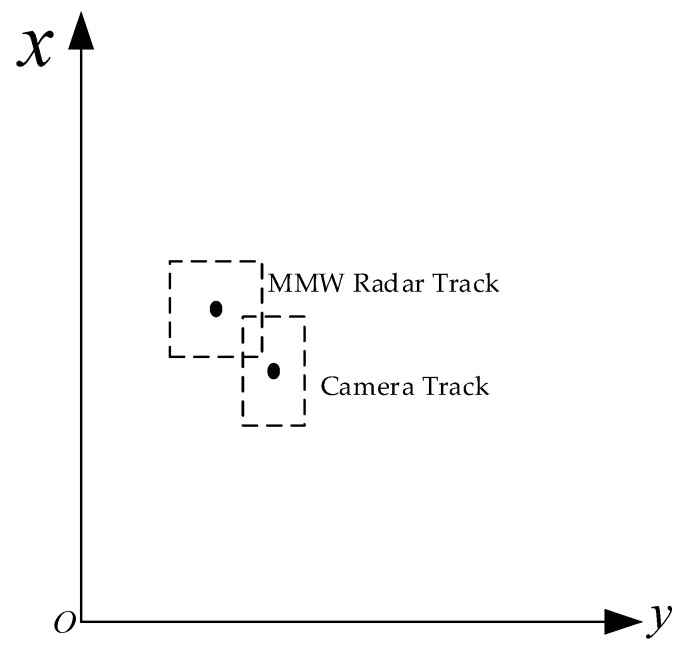
Schematic diagram of an uncertain area.

The selection of the position threshold is related to the state uncertainty of the local track, which is expressed by the state covariance in the state estimation process. In this paper, the rotation in the target motion process is ignored, and the rectangular uncertain region is established with the current local track position as the center, as shown in Figure 4. The length and width of the uncertain region are related to the position standard deviation of the local track in the longitudinal and lateral directions, respectively. For the local track, *i*, with state *X* and state covariance *P*, the length and width of the uncertain region are, respectively, as follows

(9) $$Length=2K_{GL}\sqrt{P[0][0]},$$

(10) $$Width=2K_{GW}\sqrt{P[3][3]},$$

where $K_{GL}$ and $K_{GW}$ are the constants. Because there is a two-level association, the first-level association can select a smaller threshold to ensure that the association is valid.

Regional collision association means that if two local tracks belonging to different sensors intersect with their uncertain regions, the association of two local tracks can be determined. The pseudocode to execute the regional collision association is shown in Algorithm 1.
**Algorithm 1** Regional Collision Association1:**if**$\left|X_{i}.x-X_{j}.x\right|>K_{GL}\cdot sqrt\left(P_{i}\left[0\right]\left[0\right]\right)+K_{GL}\cdot sqrt\left(P_{j}\left[0\right]\left[0\right]\right)$**then**2:**return** false3:**else if**$\left|X_{i}.y-X_{j}.y\right|>K_{GW}\cdot sqrt\left(P_{i}\left[3\right]\left[3\right]\right)+K_{GW}\cdot sqrt\left(P_{j}\left[3\right]\left[3\right]\right)$**then**4:**return** false5:**return** true

In order to quantify the degree of association between two local tracks, the concept of the Jaccard coefficient is cited. The Jaccard coefficient refers to the ratio between the intersection and union of the two sets. Here, the uncertain regional area of the local track is used to refer to the set, and the expression can be obtained as follows

(11) $$J=\frac{\left|S_{r}\cap S_{c}\right|}{\left|S_{r}\cup S_{c}\right|}=\frac{\left|S_{r}\cap S_{c}\right|}{\left|S_{r}\right|+\left|S_{c}\right|-\left|S_{r}\cap S_{c}\right|},$$

where *J* is called the association similarity index, which represents the association degree of two local tracks. $S_{r}$ and $S_{c}$ represent the uncertain region area of the local tracks of the MMW radar and camera, respectively.

*FusionLife* is set up to indicate the stability of the fusion track. When the fusion track is initially formed, the *FusionLife* value is 0. If the local tracks corresponding to the fusion track are all associated in the subsequent continuous period, then the *FusionLife* value is accumulated. When *FusionLife* reaches the set threshold of *FusionLifeMax*, it indicates that the fusion track is relatively stable. Then, in the later fusion time node, the corresponding local tracks that do not need to participate in the association algorithm can be directly used to update the state of the fusion track. If the *FusionLife* is less than *FusionLifeMax* at a certain fusion time node, and the corresponding two local tracks are not associated, the fusion track will die out.

For the fusion track obtained through regional collision association, the *FusionLife* value accumulation mode is as follows

(12) $$FusionLife=FusionLife+\mu_{area}•J,$$

where $\mu_{area}$ is a constant coefficient.

#### 4.1.3. Weighted Track Association

It is assumed that for an MMW radar and a camera, there are local tracks, *i* and *j*, respectively. Through previous tracking processing modules, the state estimation of two local tracks is ${\hat{X}}_{i}(k|k)$ and ${\hat{X}}_{j}(k|k)$, and their state covariance is $P_{i}(k|k)$ and $P_{j}(k|k)$. The state estimation difference of two tracks is expressed as follows:

(13) $$\Delta {\hat{X}}_{ij}(k|k)={\hat{X}}_{i}(k|k)-{\hat{X}}_{j}(k|k),$$

The null hypothesis and alternative hypothesis are established, and the track association problem is transformed into a hypothesis testing problem.

$H_{0}$: ${\hat{X}}_{i}(k|k)$ and ${\hat{X}}_{j}(k|k)$ are the track state estimation of the same target, namely, track *i* and *j* are associated;

$H_{1}$: ${\hat{X}}_{i}(k|k)$ and ${\hat{X}}_{j}(k|k)$ are not the track state estimation of the same target, namely track *i* and *j* are not associated.

It is assumed that the state errors for the local tracks of the same target are statistically independent. Under the $H_{0}$ assumption, the state estimation differences covariance of track *i* and *j* can be expressed as

(14) $$C_{ij}(k|k)=E[\Delta {\hat{X}}_{ij}(k|k)\Delta {\hat{X}}_{ij}^{T}(k|k)]=P_{i}(k|k)+P_{j}(k|k).$$

The statistical value of weighted track association is as follows

(15) $$\alpha_{ij}(k)=\Delta {\hat{X}}_{ij}^{T}(k|k)C_{ij}(k|k)\Delta {\hat{X}}_{ij}(k|k).$$

Under the $H_{0}$ assumption, the state estimation difference $\Delta {\hat{X}}_{ij}(k|k)$ obeys gaussian distribution, and the statistical value $\alpha_{ij}(k)$ obeys chi-square distribution. The chi-square distribution association threshold γ is selected. When $\alpha_{ij}(k)$ is less than γ, the hypothesis $H_{0}$ is accepted, and tracks *i* and *j* are considered to be associated. Otherwise, we accept the hypothesis that tracks *i* and *j* are unassociated.

For the fusion track obtained through the weighted track association, the *FusionLife* value is accumulated in the form of

(16) $$FusionLife=FusionLife+\xi_{Weight},$$

where $\xi_{Weight}$ is a constant. The associated quality is not evaluated here, so only a set constant $\xi_{Weight}$ is used as the added value of *FusionLife*.

### 4.2. Fusion Track State Estimation

**Figure 5:**
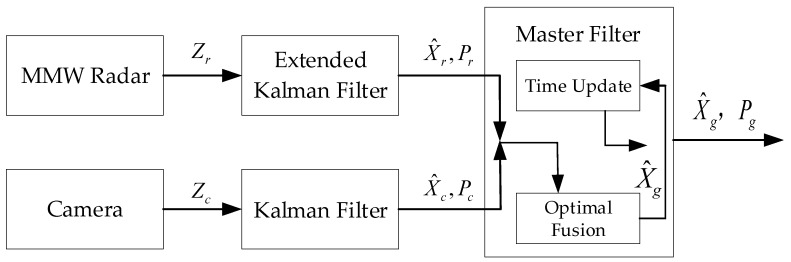
Fusion track state estimation.

A federated filter is applied to the distributed fusion structure [25], which can be used to build the connection of the state estimation part between the tracking processing modules and the fusion center module. A federated filter can be generally divided into four basic structures, namely: fusion-reset mode, zero-reset mode, no-reset mode, and rescale mode. In the non-reset mode, there is no information reset from the master filter to the sub-filters, so the sub-filters will not pollute each other. The non-reset mode is fast in computation and strong in fault tolerance. This paper designs a fusion track state estimation method based on the non-reset federated filter structure, as shown in Figure 5. The figure only estimates the fusion state for a single target, where $z_{r}$ and $z_{c}$ are the radar measurement and camera measurement associated with the target, respectively. ${\hat{X}}_{r},P_{r}$ and ${\hat{X}}_{c},P_{c}$ represent the state estimation output of the two sub-filters, and ${\hat{X}}_{g},P_{g}$ represent state estimation output of the master filter, which is also the state information of the fusion track. The extended Kalman filter corresponds to the state estimation of the MMW radar track, and the Kalman filter corresponds to the state estimation of the camera track. They have been designed in the tracking processing module.

The workflow of the federated filter includes the initial information determination, information allocation, time update, measurement update, and information fusion. Among them, the time update and measurement update of the two sub-filters belong to the state estimation part of the tracking processing modules, which will not be detailed here.

The target motion model adopted by the MMW radar and the camera is the same, and the target state format output by the sub-filters is the same. Therefore, the output state estimation information of the two sub-filters can be integrated into the master filter. At the initial time of fusion, the system needs to determine the initial information.

The global estimation error covariance $P_{0}^{g}$ and system process noise $Q_{0}^{g}$ at the initial moment can be calculated from Equations (17) and (18).

(17) $$P_{0}^{g}={\left[{\left(P_{0}^{r}\right)}^{-1}+{\left(P_{0}^{c}\right)}^{-1}\right]}^{-1},$$

(18) $$Q_{0}^{g}={\left[{\left(Q_{0}^{r}\right)}^{-1}+{\left(Q_{0}^{c}\right)}^{-1}\right]}^{-1}.$$

For a non-reset federated filter, the information is allocated only at the initial time. The initial information is generally distributed evenly. However, because of the different measurement accuracies of the millimeter wave radar and camera, if the initial information is distributed equally, the global estimation accuracy will be reduced. Now, $\Lambda_{r}$ and $\Lambda_{c}$ are set to represent the sum of the state covariance singular values of two sub-filters, respectively, and the singular values are used to calculate the two information allocation coefficients.

(19) $$\beta_{0}^{1}=\frac{\Lambda_{r}}{\Lambda_{r}+\Lambda_{c}},$$

(20) $$\beta_{0}^{2}=\frac{\Lambda_{c}}{\Lambda_{r}+\Lambda_{c}}.$$

$\beta_{0}^{1}$ and $\beta_{0}^{2}$ represent the initial information allocation coefficients of the millimeter wave radar and camera local tracks, respectively. The coefficients are used to assign information about sub-filters and to update their initial information

(21) $$\begin{array}{cc} \{\begin{array}{c} Q_{0}^{i}=\beta_{i}^{-1}Q_{0}\\ P_{0}^{i}=\beta_{i}^{-1}P_{0}^{g}\\ {\hat{X}}_{0}^{i}={\hat{X}}_{k}^{g} \end{array} & i=1,2 \end{array}.$$

where i=1 represents the local track information of the millimeter wave radar, i=2 represents the local track information of the camera. In the information fusion part, the local state estimation information obtained by two independent sub-filters is fused to obtain the global optimal estimation

(22) $$P_{k}^{g}={\left[{\left(P_{k}^{r}\right)}^{-1}+{\left(P_{k}^{c}\right)}^{-1}\right]}^{-1},$$

(23) $$X_{k}^{g}=P_{k}^{g}\left[{\left(P_{k}^{r}\right)}^{-1}{\hat{X}}_{k}^{r}+{\left(P_{k}^{c}\right)}^{-1}{\hat{X}}_{k}^{c}\right].$$

## 5. Experimental Result

**Figure 6:**
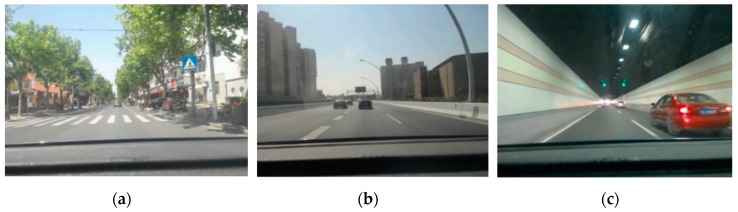
Experimental environment: (**a**) street; (**b**) expressways; (**c**) tunnels. They, in turn, correspond to the following three sets of experimental data.

In this paper, the ego vehicle was equipped with a Delphi’s multimode electronically scanning radar (ESR) and a camera with a Mobileye Q3 chip. The MMW radar was installed in the middle of the front bumper of the ego vehicle, and the camera was installed in the windshield inside the longitudinal symmetry plane of the ego vehicle on the side of the cab. The inertial navigation system was also installed to detect the ego vehicle status. The sensor fusion experiment was carried out in an urban road environment, including street, expressways, tunnels, etc., as shown in Figure 6. Several typical working conditions were selected from the experimental data for the analysis.

### 5.1. Single Target Fusion

**Figure 7:**
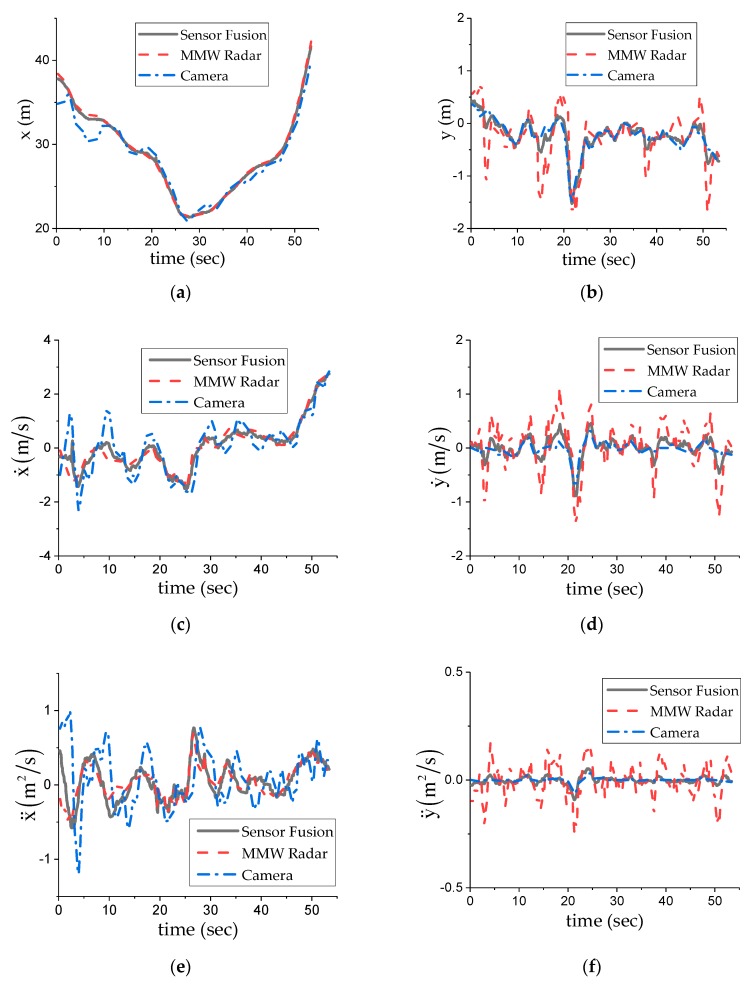
Experimental results of single target fusion: (**a**) longitudinal distance; (**b**) lateral distance; (**c**) longitudinal relative velocity; (**d**) lateral relative velocity; (**e**) longitudinal relative acceleration; (**f**) lateral relative acceleration. In the figure, MMW radar curve and camera curve refer to the state estimation of local track, while sensor fusion curve refers to the state estimation of fusion track.

In Figure 6a, there is only one vehicle target, which is detected by two sensors, and the fusion algorithm is run. Figure 7 shows the fusion experiment results. The whole experiment was divided into two sections, with a boundary of 28 s. The first section was gradually close to ego vehicle, while the second section was gradually away from ego vehicle, as the speed increased. During the whole experiment, the millimeter wave radar track was more stable in a longitudinal direction, and the camera track was more stable in a lateral direction, which is consistent with the characteristics of the two sensors. After the fusion algorithm, the state information of the fusion track was obtained. The longitudinal information was more like the MMW radar, and the lateral information was more like camera. The fusion track information was relatively stable and smooth, except for most of the spikes of the sensor track. Within a period of 0~10 s, the two local tracks had a relatively large deviation in the longitudinal distance, but from the perspective of all of the state components, their motion trend was the same. According to the weighted track association, they can be associated with the same target. After 10 s, the positions of the two local tracks are similar, and the association can be realized through the regional collision correlation. With the passage of time, the fusion track becomes stable gradually, and the corresponding local tracks can be fused directly, without any association.

### 5.2. Multi-Target Fusion

**Figure 8:**
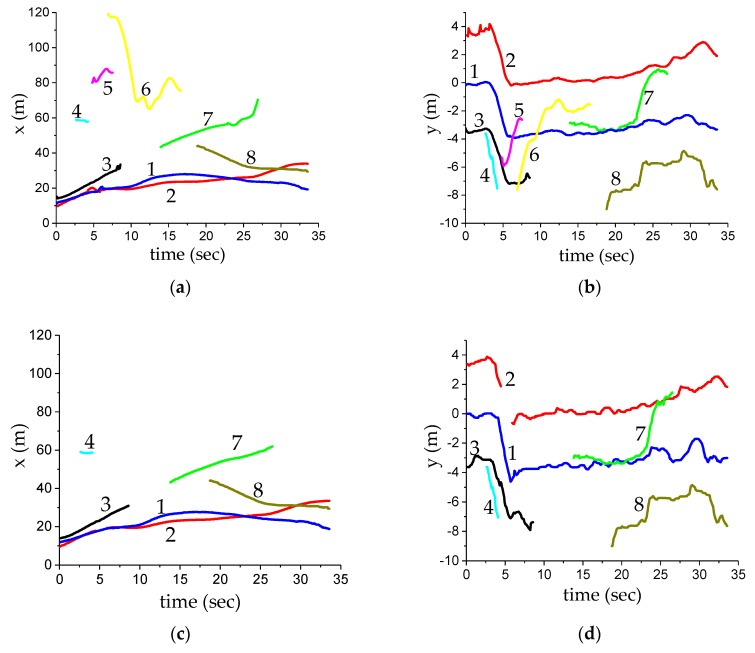

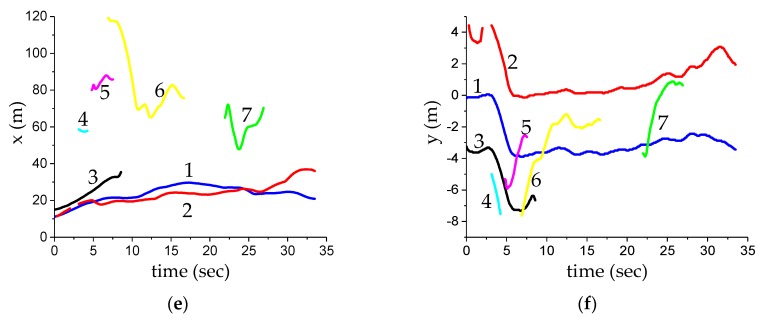
Experimental results of the multi-target fusion: (**a**) longitudinal distance of global tracks; (**b**) lateral distance of global tracks; (**c**) longitudinal distance of MMW radar tracks; (**d**) lateral distance of MMW radar tracks; (**e**) longitudinal distance of camera tracks; (**f**) lateral distance of camera tracks.

Figure 6b shows the multi-target motion condition on the urban expressways. Figure 8 shows the fusion experiment results under the multi-target condition. The ego vehicle was originally in the middle lane, it changed to the right lane after around five seconds, and then kept driving along the straight line. Each target track in the figure is represented by a curve of different colors and is marked with a serial number for convenient analysis. After about 35 s, the radar detected six targets, the camera detected seven targets, and the fusion algorithm confirmed the existence of eight targets. Among them, targets numbered 1, 2, 3, 4, and 7 were detected by two sensors, and the No. 8 target was detected only by the MMW radar, and the No. 5 target and No. 6 target were detected only by the camera. For the No. 2 target, the camera tracking failed within 2 to 3 s, and the MMW radar tracking failed within 4.5 to 5.7 s. Therefore, the local track information of the MMW radar and camera was used in the early stage, and then it became the fusion track. For the No. 7 target, only the MMW radar was tracking within 13.5~22 s, and the camera formed a confirmed track in 22 s, and the two were associated into a fusion track. As the distance is relatively far and is affected by the light, the camera had a large error in the longitudinal distance. At the initial stage of the fusion, the detection position of the camera and MMW radar was still close, and they could be linked together through the regional collision association. When there was a large error in camera detection, the corresponding two local tracks could be directly used for fusion due to the setting of the fusion track stability. The No. 4 and No. 5 targets appeared for a short time because of the occlusion between the targets.

### 5.3. Application of Sensor Fusion

We refer to the targets closest to the ego vehicle in each lane as the dangerous targets, which have a significant impact on the decisions of the intelligent vehicle control system. Adaptive cruise control and autonomous emergency braking subsystems need to know in a timely manner whether there are dangerous vehicles in front of the ego vehicle, including dangerous vehicle targets such as a vehicle in the main lane in which the ego vehicle is located, and a vehicle cut in from the side lane [26]. This paper presents an example of dangerous target selection in the presence of target cut in and cut out in the main lane, as shown in Figure 6c. The same method can also be used to screen the dangerous targets of side lanes.

**Figure 9:**
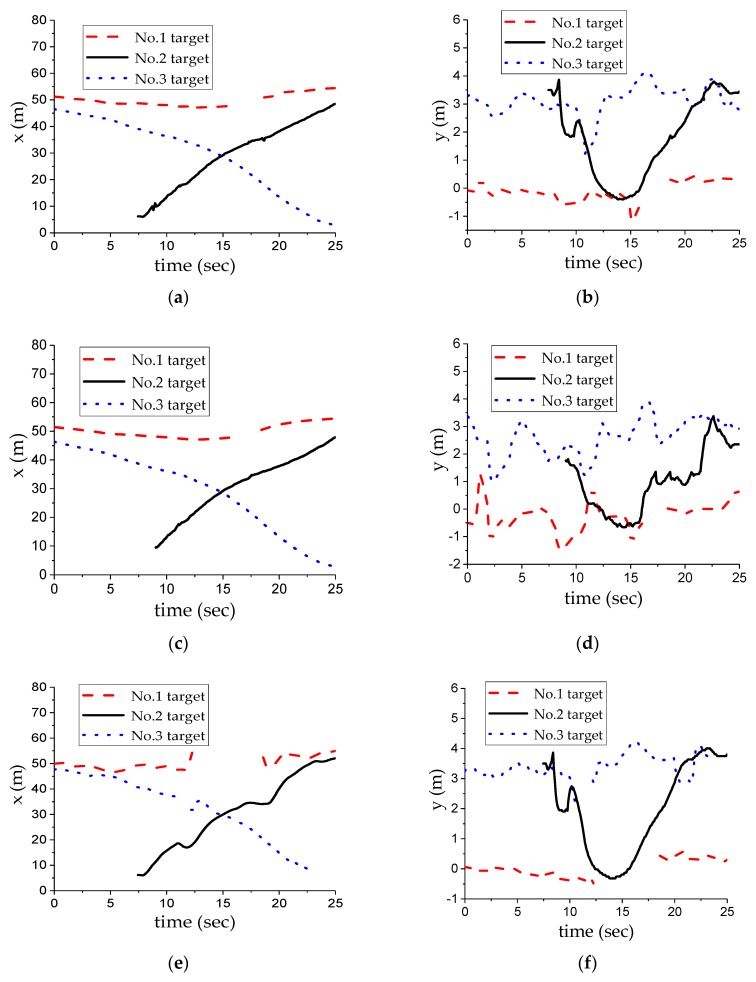
Experimental results of overtaking conditions. The meaning of each coordinate diagram is the same as that in Figure 7.

In the experiment results shown in Figure 9, the No. 1 target was driving in the main lane, and the No. 3 target was driving in the right lane. The No. 2 target was detected to be in the right lane at 7.4 s, then it cut into the main lane, and cut back into the right lane after passing the No. 3 target. During the overtaking period, the No. 2 target obscured the No. 1 target, and the detection performance of the MMW radar was better than that of the camera. Therefore, the MMW radar tracks can be used to maintain the target information, which also shows an advantage of sensor fusion. When targets were detected by both sensors, fusion tracks showed a better comprehensive performance after the fusion algorithm. With the help of the lane information provided by the camera, we could accurately judge the cutting in and cutting out time of the No. 2 target and timely change the dangerous target of the main lane.

**Figure 10:**
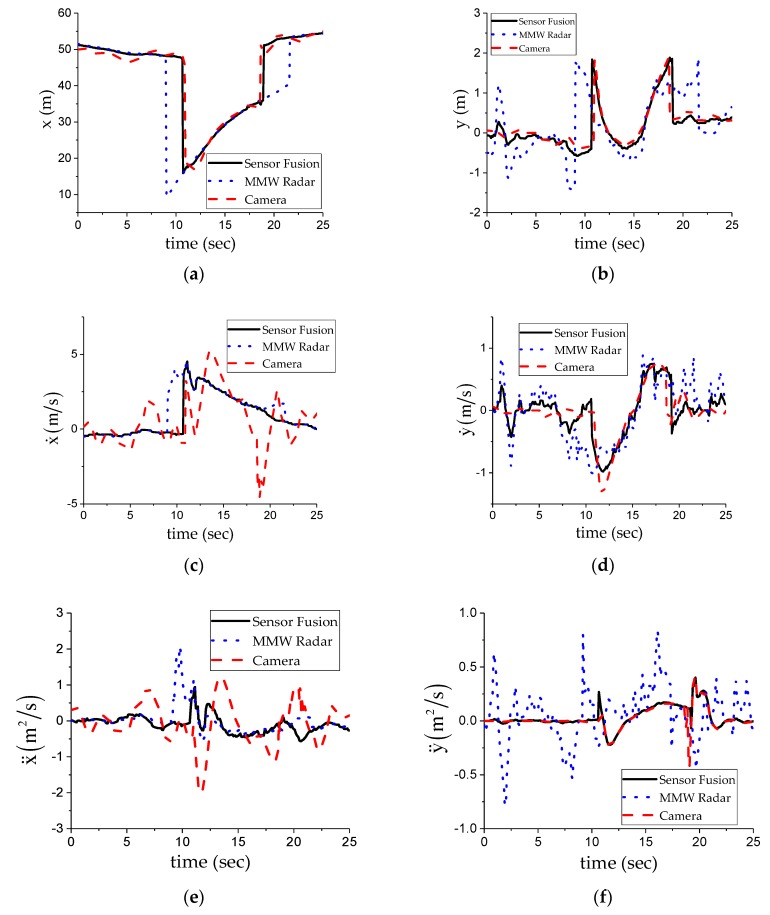
State estimation results of the dangerous target. The meaning of each coordinate diagram is the same as that in Figure 7.

Figure 10 shows the dangerous target state obtained using an MMW radar, camera, and fusion target, respectively. Compared with a single sensor, the fusion target provides more accurate state information. By playing back the collected video, we can see that the switching time of the dangerous target after fusion processing is more consistent with the actual situation. After the fusion algorithm, the dangerous target state curve is more stable, which can provide more accurate target state information for the control system.

## 6. Conclusions

In this paper, an algorithm framework of target level fusion of an MMW radar and a camera is designed. Combined with the regional collision association and weighted track association, a two-level structure is proposed for local track association. Based on the non-reset federated filter, the state estimation of the fusion track is completed. In this paper, the single-target fusion, multi-target fusion, and the application of sensor fusion in dangerous target screening are selected. In all of the experiments, the association for different local tracks of the same target is good, and the overall performance of the fusion track state estimation is better than that of a single sensor. In the experiment of selecting dangerous targets, the fusion algorithm can replace dangerous targets more accurately and timely. In the future, we can consider using more accurate sensors to detect target state information and take it as the reference value of the truth value, so as to quantitatively analyze the accuracy of a fusion track.
